# The Allocation of Attention to Learning of Goal-Directed Actions: A Cognitive Neuroscience Framework Focusing on the Basal Ganglia

**DOI:** 10.3389/fpsyg.2012.00535

**Published:** 2012-12-21

**Authors:** E. A. Franz

**Affiliations:** ^1^Division of Science, Department of Psychology, University of OtagoDunedin, New Zealand

**Keywords:** focused attention, basal ganglia, split-brain, bimanual actions, allocation of attention

## Abstract

The present paper builds on the idea that attention is largely in service of our actions. A framework and model which captures the allocation of attention for learning of goal-directed actions is proposed and developed. This framework highlights an evolutionary model based on the notion that rudimentary functions of the basal ganglia have become embedded into increasingly higher levels of networks which all contribute to adaptive learning. Supporting the proposed model, background literature is presented alongside key evidence based on experimental studies in the so-called “split-brain” (surgically divided cerebral hemispheres), and selected evidence from related areas of research. Although overlap with other existing findings and models is acknowledged, the proposed framework is an original synthesis of cognitive experimental findings with supporting evidence of a neural system and a carefully formulated model of attention. It is the hope that this new synthesis will be informative in fields of cognition and other fields of brain sciences and will lead to new avenues for experimentation across domains.

## Introduction

William James famously stated “*Everyone knows what attention **is**. It is the taking possession by the mind, in clear and vivid form, of one out of what seem several simultaneously possible objects or trains of thought. Focalization, concentration, of consciousness are of its essence*” (James, 1890/1918, p. 403–404; italics and bold added). But, what is attention ***for***? The present paper builds on the idea that attention is largely in service of our actions. What follows is the development of a framework and model of attention-for-action derived from experimental evidence based on studies in the so-called “split-brain” (surgically divided cerebral hemispheres) and related studies on fundamental processes of the basal ganglia, with a key focus on bimanual actions. A multilevel cognitive-neural system of attention is built upon processes of a highly adaptive basal ganglia-thalamic-cortical system.

Historically, studies on attention and conscious experience have focused very much on the stimulus end of processing. One might wonder whether our working definitions of attention might have evolved differently had the focus been closer to the “other end,” an issue raised many years ago by Neisser (1967). In a related vein, Neumann (1987) considered focused attention in close relation to action selection using a simple illustration of a person selectively reaching for an apple from a tree, in the context of many other possible actions (or reaching movements to other apples) which could have been performed (Franz, 2006). In a parallel development, others have considered sensory effects as virtually the same as the motor behavior associated with their occurrence, with a modified notion of “ideomotor compatibility” forming the basis of recent accounts in which “stimulus” and “response” are conceptualized as reflecting one and the same perceptual representation (Hommel, 2010). The present account is consistent with these parallel developments, and began with the basic question: *what is the relation between attention and action?* In delving into the literature, some prescient insights of early scholars who probed the nature of this relationship became apparent, and a second rather unavoidable question also emerged: *what is the relation between attention and conscious experience?* The present paper begins with a brief recapitulation of those early insights. Using these two key questions as guides, it follows with a brief review of initial experimental studies on the “split-brain” and evidence from more recent experimentation including studies from our own lab, culminating into the proposed multilevel framework of attention which aims to address the first question in detail, while also shedding some light on the second one. I first provide some working definitions, clarification of the present scope, and a summary of the proposed framework that will then be elaborated along with supporting evidence.

Although consciousness is not the topic of this paper, its derivative (i.e., conscious experience) is referred to, which I define as an organism’s dynamic awareness of stimulation, which may be associated with sensations, perceptions, thoughts, feelings, and emotions. In my view, under normal circumstances most humans possess the capacity for conscious experience and attentive behavior (ala William James) and this can also be said of animals, at least some of them, albeit to differing degrees. It is beyond the present scope to attempt to define the constraints of this capacity in the animal kingdom, or the difference in degree to which humans and non-human animals possess consciousness. Importantly, attention evolved through millions of years of adaptive changes in a dynamic environment, and in my view, experimentation is crucial in order to capture and ultimately understand critical principles pertaining to time-slices of this process. Furthermore, in embracing the basic tenets of evolution my view is that the brain evolved in an embodied manner through interactions with the environment, which also implicates an embodied view of cognition (Franz, 2010; Franz and McCormick, 2010; Franz and Gillett, 2011).

**Figure 1:**
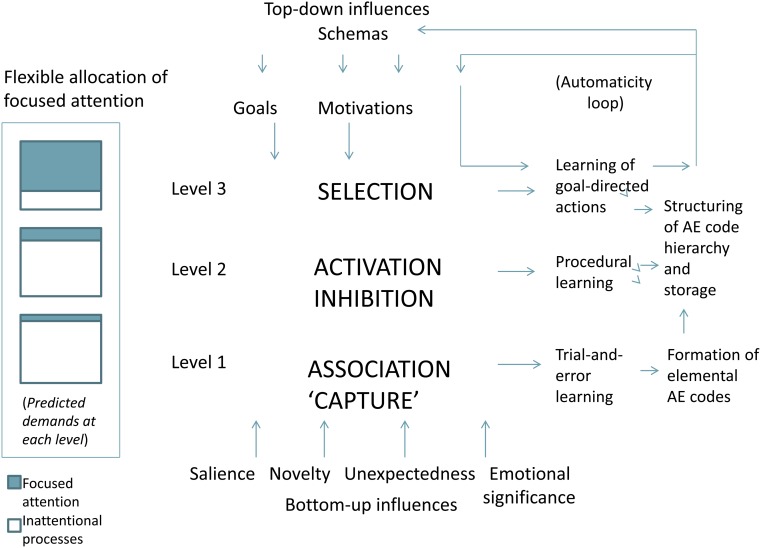
**The proposed framework underlying an allocation of attention system, presented in summarized form**. Key processes are highlighted in bold caps at each of the three conceptualized levels of the system, and arrows indicate general direction of information flow, although all levels are highly interacting with many feedback projections not shown herein. An automaticity loop is shown in the upper right corner as an adaptive module built primarily upon level 3 of the system. In the far left panel is a depiction of the approximate proportion of focused attention versus inattentional processes predicted to be involved at each conceptualized level. AE, action-effects. See text for details.

This framework highlights an evolutionary model based on the notion that rudimentary brain functions have become embedded into increasingly higher (i.e., more highly embedded) levels of networks, with evolution reflected in phylogeny, ontogeny, and adaptive learning. A multilevel attention system is summarized herein in terms of three conceptualized levels (Figure 1): Level (1) involves primarily exogenous inputs which essentially capture focused attention transiently when environmental stimuli are salient, unexpected, novel, and/or have emotional significance, so that rudimentary forms of learning first occur in a trial-and-error manner, with eventual associations between sensory effects and motor movements forming elemental action-effects perceptual codes; Level (2) reflects more elaborated forms of endogenous attention in which processes of activation and inhibition enable for sequencing and switching between available action-effects codes (and procedural learning); and Level (3) which is further dependent on endogenous (focused) attention performs a selection-like function (built upon the rudimentary forms of association, activation, and inhibition at lower levels) while receiving strong influences of top-down effects related to prior experiences associated with schemas, goals, and motivations. These processes result in a hierarchy of perceptual codes with the highest levels representing goal-related actions, and also contributing to an “automaticity loop” in which expertise and habits result through extensive learning. By “higher” it is meant that each level builds upon and embeds the so-called “lower” ones; thus, there are no strict boundaries of these conceptualized levels, with higher levels embedded upon and elaborating processes of lower levels.

It is proposed that all levels depend critically on a highly adaptive basal ganglia-thalamic-cortical system of which dopamine is a key neurotransmitter (among others). The different levels do not uniquely point to conscious or unconscious processes (Lamme, 2003), nor does the involvement of focused attention dictate any particular mix of conscious versus unconscious processing. However, it is proposed that increasingly higher levels of processing evolved with greater reliance on cortical networks; thus, the higher level networks are built upon (i.e., evolved from) the more rudimentary (subcortical) processes of the basal ganglia and their feedback loops with the thalamus. Nonetheless, even lower levels likely involve some cortical inputs, even though intrinsic circuits of the basal ganglia, together with feedback connections with the thalamus, make up the basic functions of the lower levels of the system. Key processes ascribed to these levels and their rudimentary forms are described as “*focused attention capture*” *and association* (Level 1), *activation and inhibition* (Level 2), and *selection* (elaborated forms of the combined effects of capture, association, activation, and inhibition derived from lower levels).

The proposed framework has some overlap with specific features of existing models in the literature, which I will attempt to point out where known. However, to my knowledge, the formation of a system of *allocation of focused attention* built upon the key proposed processes (capture, association, activation, inhibition), in combination with a neural model describing an adaptive basal ganglia-thalamic-cortical system, is original to this framework. Two additional classes of constraints (not illustrated in Figure 1) will be referred to as (1) *response dependencies*, defined as stimulus or response constraints that limit the availability of information to a processing network, and (2) *unifying constraints*, which condense the information that is available within a processing network. Both are described as influences on (and within) the proposed system. The latter is viewed as the result of the brain’s in-built propensity at all processing levels to unify information into coherent perceptions, thoughts, and actions, something that has been described metaphorically as reflecting forms of conceptual binding, with a loose analogy to binding properties in the brain (Franz, 2010; Franz and McCormick, 2010). In sum, the proposed multilevel system involves highly evolved networks that flexibly allocate focused attention, ultimately shaping our conscious experience.

## Back to First Principles: Some Prescient Insights

In the 1950s Roger Sperry suggested an utmost challenge when he stated “An analysis of our current thinking will show that it tends to suffer generally from a failure to view mental activities in their proper relation, or even in any relation, to motor behavior. The remedy lies in further insight into the relationship between the sensori-associative functions of the brain on the one hand and its motor activity on the other” and furthermore, “*the entire output of our thinking machine consists of nothing but patterns of motor coordination*” (Sperry, 1952, p. 291). These ideas, which likely were influenced by the behaviorism prevalent at the time, evolved later into Sperry’s growing interest in mentalism, a pursuit of his for nearly three decades following. But his statements about *sensori-associative functions* and *motor behavior* struck me as key clues that should be explored further.

The idea that the brain is a highly embedded sensorimotor machine dates back to the writings of John Hughlings Jackson based on his personal observations of clinical cases with aphasia and epilepsy among other random conditions, and informed by evolutionary theory as espoused by his contemporary, Herbert Spencer. Hughlings Jackson’s (1884) basic claim was that the brain is a large sensorimotor machine, with embedded levels of sensorimotor representations, and with higher levels overriding and inhibiting lower levels. This idea grasped the imagination of scholars in Psychology, most notably that of William James who referred to “psychical evolution” in terms of simple sensory and motor elements, clearly acknowledging Hughlings Jackson for the explanation that epileptic seizures capture “the loss of consciousness…” due to the most highly organized brain processes being exhausted and thrown out of gear…” (James, 1890/1918; Vol. 11, pp. 125–126).

Although in days of behaviorism, it was primarily the “stimulus-to-motor-response” that was conceived as the general direction in which sensory stimuli could influence behavior, further experimental evidence and thought has led people to consider the *sensory effects of an action*, whether they be kinesthetic (as suggested by William James) or derived from other sensory modalities such as audition and vision, as becoming part of a perceptual code for an action. This view most closely resonates with the work of Bernhard Hommel, and is embraced in the present paper. To illustrate the notion of “sensory effects” or “sensory consequences” consider for example, a low level organism with its basic response mechanisms. It responds to the environment, as in the photo response of the flagella in multicellular volvocine algae (Solari et al., 2011). People might argue about whether or not the organism is “aware” of the *consequences or effects* of such responses (Thorndike, 1913), but most would agree that with evolution came an increasing tendency for animals to take note of, or become aware even in a most rudimentary manner, that a response brings food or the avoidance of pain (both of which can be considered *sensory effects*). One might even argue that minimal levels of awareness brought about the development of elemental units of knowledge, and eventually increasing levels of learning, with memories formed in a Hebbian (Hebb, 1949) sort of way. Indeed, the capacity for decision-making about whether or not to respond in the same manner depending on the internal or external milieu after millions of years of evolution has become a hallmark of human behavior.

Referring again to Sperry’s earlier views, although articulated in a manner that suggested independence from those of Hughlings Jackson, they also clearly reflected a firm grounding in evolutionary theory and underlying sensorimotor processes, dating back to his lucid descriptions of animals lower on the vertebrate scale (i.e., salamanders and lower fishes) as having a nervous system concerned primarily with motor activity, with sensation and perception largely serving the guidance of responses. Sperry further suggested that this should be similar in man, given that the operating principles are fundamentally the same, and that “Cerebration, essentially, serves to bring into motor behavior additional refinement, increased direction toward distant, future goals, and greater over-all adaptiveness and survival value” (Sperry, 1952, p. 299). While I also embrace this view, I would further clarify his reference to “cerebration” as *cortical-subcortical networks and circuits*, emphasizing research from more recent decades which I will argue, strongly implicates subcortical interactions as essential to a system which allocates our attention to action, in which the basal ganglia and their connective circuits with the cortex, and loops with the thalamus are of primary importance. Notably, other subcortical structures such as the cerebellum and hippocampus are undoubtedly also involved, but will not be highlighted in the present paper.

## Original Split-Brain Experiments in Humans: A Platform for the Present Account

Key clues about focused attention, and also *unifying constraints* and *response dependencies* (to be explained further), stem from initial investigations into the so-called “split-brain.” The medical puzzle of how to ameliorate the bilateral spread of seizures associated with intractable epilepsy led to the procedure which involves surgical separation of the left and right sides of the cerebral forebrain commissures. Performed only in rare cases of severe seizures in an attempt to prevent their rapid spread between the two cerebral hemispheres of the brain this procedure also led to remarkable examples of generosity on the part of surgical patients willing to participate in experiments which eventually would completely revise scientific understanding of the human brain. Commissurotomy, one form of split-brain, involves surgical separation of an estimated 200 million direct interhemispheric white matter tracts comprising the corpus callosum, in addition to the anterior commissure and the posterior (often called hippocampal) commissure, whereas callosotomy refers to the surgical separation of the corpus callosum alone (Levy et al., 1972). In some references these terms are used interchangeably, but inappropriately so, even though the functional consequences may be similar in some contexts.

Myers and Sperry (1958) noted that clinical neurologists observed intellectual deficits in patients of these procedures while also pointing out the curious disagreement that “actual observations on the effect of complete surgical section or of total agenesis of the corpus callosum in man and other animals have indicated a surprising absence of deficit” (citing some well-known sources including Bruce, 1890; Akelaitis, 1944; Bridgman and Smith, 1945), clearly revealing that further investigation was necessary in an attempt to solve this paradox. However, Sperry’s initial writings on the split-brain suggest that his own experiments involved cats or rhesus monkeys (Glickstein and Sperry, 1959, 1960; Schrier and Sperry, 1959), not humans.

Our understanding of the “split-brain” in humans took a great leap forward with initial studies led by Michael Gazzaniga using clever techniques of lateralization including tachistoscopic presentation in the case of vision and stereognosis in the case of manual perception. Initial visual studies on humans with commissurotomy involved presenting images in the form of light flashes, letters, or words, to the left and/or right visual fields separately using tachistoscopic presentation to attempt to ensure separation of input to the two hemispheres. Those tests reinforced a most interesting finding, that the two visual fields are mapped to separate cerebral hemispheres, with the left visual field mapped to the right hemisphere and the right visual field mapped to the left hemisphere, illustrating a disconnection (also called “deconnexion”) of visual fields (Gazzaniga et al., 1962).

W.J., one of the three commissurotomy participants of those initial studies, could locate points of tactile stimulation on the fingers of either hand using touches of the thumb on the same hand, even with a 5-s delay following the tactile stimulation, but could not cross-locate such points with either hand to the other despite his ability to locate points stimulated on the head, face, and upper neck with either hand (suggesting bilateral wiring of the involved systems in the latter cases). He could produce tapping movements with each hand after being tapped on that same hand by an experimenter, although when tapped on one hand he could not always copy by producing the number of taps with the opposite hand (Gazzaniga et al., 1962, 1963). Those findings further pointed to a lateralization of both input and output of the distal manual extremities, with each cerebral hemisphere receiving primary input from the contralateral hand/fingers, owning some representation (memory) of that hand’s interactions with the environment, and projecting motor commands to that same (contralateral) hand. Decades of further study further suggested in other commissurotomy patients that the two hemispheres cannot directly compare visual or tactile stimulus information presented in a lateralized manner (Reuter-Lorenz et al., 1995), contributing to the host of evidence revealing disconnection in both visual and manual modalities. These examples illustrate important *response dependencies* which limit the availability of information to a particular system, or network (in this case, a cerebral hemisphere), further influencing the system of attention allocation for action.

Other clever tasks invented by Gazzaniga et al., 1963; p. 211) include presenting light taps “applied doubly, i.e., at two separate points simultaneously on opposite sides of the body, occasionally at corresponding symmetrical points, but more often at non-symmetrical points.” Although those points could be mapped and responded to in the manner described above, “when a subsequent verbal description of the two stimulus points was asked for, those on the left side could not be reported. This was true in spite of the fact that the subject had correctly found the point of stimulation with his left hand.” Thus, what was a *conscious experience*, as indicated by the left hand, could not be verbally reported (by the right hemisphere), again illustrating a response dependency and what has become perhaps the most important and influential finding in split-brain research and neurology in general (also critically informed by the work of famous neurologist, Paul Broca). Specifically, the left hemisphere of the brain houses the speech center, at least in the typical case of the majority of people who are right-handed (and the present review refers primarily to the typical case). While some manual responses could be made to information in the right hemisphere, speech responses could not, leading to the conclusion that the right hemisphere might have a level of awareness of its contents and can make responses as long as they are not via the disconnected speech hemisphere.

A complementary type of function was described by Levy et al. (1972), with the left hand showing clear problems in producing responses to verbal commands, although when presented with non-verbal aides, the left hand was later able to learn to respond appropriately (but findings were not always consistent), and marked difficulty in making left hand movements to verbal commands was also described in one of the younger patients with commissurotomy, although it later disappeared (Gazzaniga and Sperry, 1967). Evidence that shoulder, elbow, and wrist movements could be made to verbal commands with the inability specific to the left fingers, is suggestive of a problem in translating verbal commands to the highly lateralized task of hand/finger movement. A short period of mutism was also reported in two of the commissurotomy patients of Gazzaniga and Sperry (1967) perhaps suggesting some reorganization or neuroplasticity. The researchers stated, however, “that conscious awareness is commonly present in the minor as well as in the major hemisphere and that the two separate spheres of conscious experience may proceed concurrently as well as in alternation” (p. 134). Notably, even the simplest kind of sensory information presented to the right hemisphere could not be described verbally, often leading to confabulatory responses. Although presenting stimulus information to the right hand or right visual field presented no problems in drawing with the right hand or stereognostic exploration by the right hand, when stimulus material was presented to the right hemisphere via the left hand or in the left half visual field, some of those initial patients were able to make recognizable reproductions of very simple material using the left hand, particularly in the case of basic cut-outs like a circle, square or triangle, or alphabet letters, although this control by the minor hemisphere was reportedly inferior to that of the speech dominant left hemisphere via the right hand (although some increased ability was shown in later patients; Gazzaniga and Sperry, 1967).

Key findings dating back to the original studies (Gazzaniga and Sperry, 1965) revealed in patients with commissurotomy (and presumably also absent mass a intermedia) that the right and left hemispheres work separately in perceptual, cognitive, learning, and mnemonic activities, with verbal descriptions possible only in the left hemisphere, even though good comprehension still occurs in the right hemisphere as long as there was no verbal expression, and this also leaves open the possibility that some unity of function in the hemispheres occurs via sensory effects and/or their contributions to perceptual representations. This possibility is further consistent with evidence that there is understanding of spoken instructions for retrieving objects with the left hand, and the suggestion that the engrams for language comprehension are bilateral even though speech output is only normally possible from the left hemisphere (Gazzaniga and Sperry, 1965).

Perhaps illustrated best by well-known examples of J.W. made by Michael Gazzaniga which are publically available on YouTube, a word presented to the right hemisphere does not access the speech system in the left hemisphere, but after drawing a picture of what the word stands for (i.e., an object) J.W. informs his own left hemisphere speech system of what the word is. He essentially sees the answer he has drawn on the paper in front of him, and his left hemisphere is then able to access the speech response. While such examples reveal that J.W. can, in his right hemisphere, translate from a visual image to a drawn picture even when that visual image is in word form, he cannot directly access his speech output system from his right hemisphere. It would appear that all that is missing from the right hemisphere is a speech output system. On the basis of such evidence, I will later argue that the response mode availability (i.e., speech or manual) might largely shape what content of information becomes available to conscious awareness; thus, response dependencies such as these shape our conscious experience in that they limit the information accessible for conscious processing.

## Unity of Attention in the Split-Brain

The capacity of each hemisphere to respond or attend to information, either separately or in collaboration, is an issue of critical importance. Following commissurotomy each hemisphere of the brain can respond in a rapid manner by drawing a visually lateralized presentation of a shape (such as a circle or square, or a 3-sided rectangle in various orientations), and the two hemispheres can produce such drawings simultaneously via the two hands without interference in the spatial patterns, whether the presented shapes are the same or different (Franz et al., 1996; Franz, 2003); in comparison, controls with intact brains cannot draw two different shapes simultaneously without considerable spatial interference known as spatial coupling (Franz et al., 1991; Franz, 1997; Franz and Ramachandran, 1998) and substantial increases in reaction times (Franz et al., 1996; Franz, 2003) in comparison to when drawing identical shapes. Those findings have led to the inference that spatial coupling, an abstract form of planning, relies on an intact corpus callosum. The findings also make clear that the two hemispheres have some capacity to attend and respond in parallel and virtually separately from one another when the brain is “split,” particularly on a task with high ideomotor compatibility between stimuli and responses such as copying shapes. In a similar vein, the original studies by Gazzaniga and Sperry (1966) demonstrated that people with commissurotomy could perform a reaction time task with the right hand responding to a green-red color discrimination to visual stimuli lateralized to the left hemisphere while at the same time performing a reaction time task with the left hand responding to light-dark discriminations to visual stimuli presented to the right hemisphere, with each hand responding by pressing one of two buttons located in pairs on a vertically positioned screen in front of the body. Unlike the control participants who took approximately 40% longer to respond on the dual-task compared to the single task trials, people with commissurotomy took approximately the same amount of time to respond on both conditions (Gazzaniga and Sperry, 1966), further supporting the conclusion that with commissurotomy the two hemispheres can respond in parallel to different tasks when the tasks are relatively simple.

There is also evidence that on some level, the two sides of the brain work together, as is shown by a slightly modified version of the same task used in Franz et al. (1996). When the commissurotomy patients were instructed to draw continuous lines (drawing in a repeated back-and-forth or left-right manner either horizontally or vertically along a flat surface) following visually lateralized presentation of just a single line, they still produced no obvious spatial disruptions (i.e., lack of spatial coupling) yet their hands moved together in a coordinated manner in producing reciprocal cycles of lines even without explicit instructions to do so (and without specific timing demands). Thus, the onset of movements tended to be synchronous for the two hands despite the spatial uncoupling, suggesting a role of *subcortical processes* (those unaffected by commissurotomy) in unifying the coordination of the hands (Franz et al., 1996). This provided an additional hint about *unifying constraints* in general, which in my definition results in a condensing of information during processing, due to a general propensity of the brain to “bring together” different components of movements, perceptions, and thoughts into coherent wholes, rather than to process components as separate entities, in the interest of an economy of processing (Franz, 2010; Franz and McCormick, 2010).

Other important evidence can be brought to bear on the unification of attention. An earlier study by Kreuter et al. (1972) used rapid finger tapping of the index fingers during 10-s trials to probe mental processes in the two hemispheres in five people with commissurotomy and four controls. The study reported predominantly bimanual simultaneous tapping, although there were some departures from synchrony. Perhaps the most provocative findings occurred on conditions in which participants attempted to tap with the two hands synchronously while also reciting the alphabet or performing numerical calculations under different conditions of task difficulty (and due to various reasons only a small subsample could be tested on those dual-tasks). Whereas control participants demonstrated no disruptions in rhythm of the tapping movements and no errors on the dual-task situations, marked disruptions were shown in the tapping of at least one commissurotomy participant in dual-task situations involving an alphabet task of skipping alternate letters, in which her right hand or both hands actually stopped tapping during pauses in her thinking. Even more difficult tasks (i.e., attempting to recite the alphabet backward while tapping) could not be performed at all, nor could tapping be performed while doing calculations, with her right hand more severely affected than her left (although both were affected to some extent). Remarkably, however, the participant with commissurotomy continued to tap with her left hand during some spontaneous conversation, although her right hand didn’t move while she spoke (Kreuter et al., 1972). Whereas two other participants with commissurotomy were unable to be tested on the dual-tasks due to some complications in applying the methods, one other participant with commissurotomy was able to perform the dual-tasks except for some small disruptions in tapping of the left hand while performing a verbal task. Notably, the authors suggested that interference effects were minimal in controls due to the highly automatic nature of tapping which tends not to pose additional loads on attention processes, however, when using motor tasks which involve more focused attention, such as balancing a dowel rod, there tends to be disruptions in the motor task under similar dual-task conditions (Kinsbourne and Cook, 1971). Together, the foregoing evidence leads to the following summarized points: (1) allocation of focused attention is flexible, and demands on focused attention change with learning (Kreuter et al., 1972), (2) there is some capacity for focused attention in the two hemispheres simultaneously even when they are disconnected due to commissurotomy, at least for tasks with somewhat low demands (Gazzaniga and Sperry, 1966; Franz et al., 1996), and (3) focused attention is limited in capacity (Kreuter et al., 1972) and ultimately is unified (i.e., normally shared) in the two hemispheres combined.

In the proposed framework, focused attention is flexibly allocated (Franz, 2004a) and unifying constraints operate in the interest of attention-for-action, so that focused attention depends on operations of the basal ganglia-thalamic networks that form circuits with vast regions of the cortex, and activation of specific cortical areas depends on the nature of the information, task, and situation. Because of the adaptive nature of the attention system, with increasing levels of learning (which requires focused attention), expertise is achieved, and this essentially changes the allocation of attention within the (dynamic) system.

Consider for illustrative purposes, a skilled musician’s remarkable ability to produce smooth melodies despite playing different rhythms with the two hands, as in the case of pianists. Producing two different rhythms simultaneously is something most unskilled novices cannot do, which suggests that they cannot successfully divide attention between the two hands, and/or that the attention required to produce two different rhythms exceeds the limited capacity available. But with practice, aspects of the tasks become more automatic in that less focused attention is needed. Thus, expertise brings about an increased ability to focus on high level representations such as the melody, replacing the earlier (less-learned) focus on lower level sensorimotor (or elemental) representations making up the individual rhythms (Franz, 2004a). This implicates the interplay of attention, not only between the left and right sides of the brain, but also among the different conceptualized levels of the sensorimotor system (in Hughlings Jackson’s (1884) terminology), again supporting “the organism’s natural tendency to optimize actions and economize resources” (Franz and McCormick, 2010 p. 273–274; see also Franz, 2010; Franz et al., 2001).

## Influences of Response Dependencies on Availability of Information

Maintaining unified attention and coherent behavior despite separation of the two cerebral hemispheres seems at first thought impossible. But some critical clues from the early literature provide further insights. Teng and Sperry (1973) tested 6 people with commissurotomy using lateralized visual presentations or letters or digits to one or both visual fields, requiring manual responses in which a hand would select a match from a group of possible letters or digits shown in free vision on physical tiles, or verbal responses in which participants said what they saw. Despite some confusing aspects of the results which implicated the right hemisphere in speech output (which in my view cannot be firmly concluded from the data due to various task constraints and response measures), some key findings are highly relevant to the present arguments. In particular, Teng and Sperry (1973) reported evidence of extinction by the right hemisphere during tasks using bilateral stimulus presentation (in all but one participant with commissurotomy), consistent with some evidence in Kreuter et al. (1972) described above, again illustrating a response dependency. Moreover, according to Teng and Sperry (1973) other experiments revealed that even when presenting stimuli to the right hemisphere 20 ms ahead of the left hemisphere, or forcing participants to respond to the stimulus in the right hemisphere first, such extinction persisted. The authors also pointed out that when presented with other types of stimuli, such as pictures or spatially aligned letters in a row across midline (as opposed to single letters), there was less evidence of extinction in the right hemisphere, although some evidence of continuous suppression of the right hemisphere has also been shown (Teng and Sperry, 1973).

According to Kreuter et al. (1972) one hemisphere appears to withdraw attention from the task it governs when the other hemisphere has increasing demands due to task difficulty, in line with their key finding that the tapping of one or both hands is arrested during increasingly difficult levels of the dual task. This suggests that when demands exceed supply, the brain’s response is not only to reduce attention to some tasks, but even to remove it entirely. Thus, the response dependency which results in one hemisphere becoming arbiter at the expense of attention in the other hemisphere depends to some extent on the specific nature of the responses –speech is dominant in this case. Indeed, Kreuter et al. (1972; p. 460) added that “…in normal life the corpus callosum, in addition to its well established information transmitting role, must also have an attention equilibratory function.”

But, there also must be a contribution of subcortical processes in the case of response dependencies; otherwise one would not find strong evidence of such dependencies in people with commissurotomy. For example, Franz (2000; reviewed in Franz, 2003) tested participants with commissurotomy or callosal agenesis (those born without a corpus callosum) using auditory (therefore bilateral) verbal instructions to “draw continuous circles with both hands at a comfortable pace” but with no further instructions as to how to coordinate the hands (Franz et al., 2002). Interestingly, whereas in the commissurotomy participants the right hand tended to lead the left in a coordinated manner and participants adopted a mirror-symmetrical mode of drawing similar to what we find in right-handed neurologically normal controls, participants with callosal agenesis tended to draw with the left hand leading the right in a coordinated manner but with both hands moving in the same direction in space. While the reasons underlying a dissociation between groups are debated (Franz, 2003) most relevant to the present context is the evidence of left hemisphere dominance in the commissurotomy group (right hand lead) and right hemisphere dominance in the callosal agenesis group (left hand lead) on those tasks. I would argue that exertion of hemisphere dominance assists in maintaining unity between the hemispheres and this response dependency relies largely on available subcortical processes.

Hemisphere dominance might bring about coherent behavior (rather than a competition between hemispheres) due to one hemisphere becoming the arbiter or leader, although it is also possible that a rapid switch of attention occurs between the hemispheres. However, in some circumstances a switching model might be less likely to hold. For example, switching is inconsistent with the observed phase differences between hands on the circle-drawing task (Franz, 2003) and might be viewed as counterproductive in terms of economy of processing (Franz and McCormick, 2010), particularly given the wealth of literature demonstrating high costs in rapid switching between tasks even in neurologically normal participants (Hayes et al., 1998; Shook et al., 2005; Franz, 2006) although those studies did not employ specific interhemispheric manipulations. We have argued that the task, situation, and hemisphere specializations determine which hemisphere is dominant at the time (Franz, 2004a, 2010) and as the evidence suggests, such dominance can be exerted in a prolonged manner in neurologically normal controls and in people with commissurotomy (Franz, 2000, 2003). This leads to the inference that such dominance is flexible to some degree, as is focused attention (Franz, 2004a,b). Consider again a skilled musician. Imagine having a discussion with her. Her left hemisphere is likely to be dominant in the task of speaking. But, ask the same musician whether she enjoys a particular melody. It is likely to be her right hemisphere that becomes dominant when she engages in enjoyment of the music. As illustrated above, evidence suggests that in people without a corpus callosum (and perhaps also without anterior and posterior commissures, as in commissurotomy) one hemisphere also assumes dominance depending on the task and situation, at least under normal (bilateral) conditions of informational input. In this manner, flexible allocation of attention and certain response dependencies (such as hemisphere dominance) might be related, as they both implicate subcortical processes.

Response dependencies come in many forms and deserve further study to fully understand. Just as those related to hemispheric specializations in which a particular response mode is all that is available, the cognitive set elicited by instructions in an experiment can result in response tendencies which limit the information that is attentively processed. Consider the brilliant demonstrations of inattentional blindness (Neisser, 1979; Simons and Chabris, 1999). Simons and Chabris (1999) instructed observers to watch videotapes of two teams of players moving around and bouncing and passing an orange basketball, with one team wearing white shirts and the other wearing black. The observers were told to keep a silent count of the total number of passes, or number of bounces (depending on condition) made by one team (white or black) and to later write down their counts. In one of the experimental conditions, after about 44 s of the task a woman was presented (in the same videotape among the players) wearing a full gorilla costume, walking through the action arena and taking about 5 s to do so, while the players in the action continued to play. Interestingly, nearly 50% of participants reportedly failed to notice the gorilla upon later interview, leading Simons and Chabris (1999) to conclude that conscious perception does not occur without focused attention. In the present view, response dependencies (in this case, due to a compliance with instructions) largely determine which information becomes part of attentive processing and also what becomes a conscious experience. Thus, response dependencies reflect a large category ranging from in-built constraints such as hemisphere dominance, to learned influences which are elicited through experimental instructions. The foregoing evidence suggests that response dependencies operate in the neurologically normal brain and are further illuminated by behavior in the split-brain, pointing to subcortical processes as being essential in their regulation. Subcortical processes are also fitting in maintaining unity of what otherwise would be a bicameral mind (Jaynes, 1976).

## Hemispheric Competition

The above examples suggest that it is possible for subcortical processes to maintain unity through response dependencies even in a brain with lateralized response networks. However, the precise interplay between interhemispheric (commissural) processes and circuits involving subcortical processes has not been defined, even in the normal brain (nor is the present paper an attempt to do so). However, one form of cortical constraint might be worthy of further mention. A proposal from early studies of Gazzaniga and Sperry (1966) was that “the neocortical commissures serve to unify the visual world… and their presence tends to prevent the two half brains from making discordant volitional decisions” (p. 261). The latter portion of the above statement resonates well with recent convergent evidence that rudimentary callosal processes operate in the interest of preventing conflicting manual response from occurring between the two hemispheres of the brain (Shen and Franz, 2005; Franz and Fahey, 2007). Those proposed rudimentary callosal processes are not present in people born without a corpus callosum and children of normal development who are younger than age 6 years due to incomplete myelination (Franz and Fahey, 2007). Moreover, if such signals were removed in the mature brain, as in the case of commissurotomy, it would be expected that some antagonistic movements (conflicting movements between the hemispheres) might occur at least occasionally. Indeed, Sperry (1966) described a person with commissurotomy who was pulling up trousers with one hand while pulling them down with the other. In my own experience, I have observed one person with very recent commissurotomy attempting to zip a sweater with one hand while her other hand interrupted with a somewhat explosive impulse in the other direction (the unzipping direction), consistent with our earlier proposal (Franz and Fahey, 2007). However, with response-dependent hemisphere dominance, such antagonistic movements would be far less likely to occur, particularly if callosally mediated signals inform one hemisphere that a response is pending in the other (as is proposed to occur in the normal brain).

So far, the evidence described herein has implicated subcortical mechanisms in the flexible allocation of attention in relation to a range of response dependencies, and also in possible unification of actions involving effector systems on the two sides of the body. The remainder of this review attempts to further describe subcortical processes as being crucial building blocks in a system which allocates attention for action, particularly during learning. That system is proposed to interact with response dependencies while also being shaped by unifying constraints which are ubiquitous at all levels of this highly complex and adaptive system.

## The Formation, Activation, and Selection of Action-Effects Perceptual Codes in the Proposed Multilevel Framework

It is critical to point out, that, whereas we can focus attention to sensory effects, most motor movements themselves are not the focus of attention. This brings us very close to the ideas of Bernhard Hommel who has also argued that “attention is a direct derivative of mechanisms subserving the control of basic motor actions” and “perceiving and acting is thus the same process, consisting of moving one’s body in order to generate particular perceptions” which further suggests that “there is no qualitative difference between the representation of a stimulus event (which includes the action that has given rise to it) and the representation of an action plan (… the action goal, that is”; Hommel, 2010, p. 123–124). Thus, it is the action-effects perceptual codes which are likely to be represented in the brain, and this allows for a reversal of the typical stimulus-to-response unidirectional framework of earlier cognitive models to one in which a sensory (effects)-to action perceptual code might underlie goal-directed actions.

Let us again consider some earlier evidence from studies in people with commissurotomy. Holtzman and colleagues reported that such participants maintained an ability to use visual information presented to one hemisphere in predicting the location of a visual cue subsequently presented to the other hemisphere, as though one hemisphere directs attention to the other. However, when the cues in the two hemispheres conflicted, there was actually a cost (in reaction time) to respond to a subsequent cue in the other hemisphere (Holtzman et al., 1981, 1984). To further examine this, Luck et al. (1994) presented unilateral or bilateral displays of visual stimuli consisting of a target item and surrounding distracters with the hypothesis that search time in a bilateral search task should be less than in a unilateral search task (by approximately half) if searching can occur in both hemispheres simultaneously, a hypothesis that would appear at odds with the findings of Holtzman et al. (1981, 1984). Whereas Luck et al. (1994) found that search time increased with set size as would be expected, the commissurotomy patients produced far faster bilateral compared to unilateral search times, approaching the “double time” prediction of searching bilateral arrays. This was not the case in controls whose search times were similar in unilateral and bilateral conditions. The findings from the patients are in line with a model of dividing attention between the perceptual tasks undertaken in the two hemispheres (without reaching a capacity limit in either) and point to the corpus callosum in unifying attention between the hemispheres in the normal brain. Thus, at first glance the findings appear inconsistent across lab groups in terms of whether or not the two hemispheres appear to be able to divide attention on visual tasks. However, this is not necessarily the case on reanalysis. The task of Holtzman and colleagues involves *directed attention to sensory cues*, which highlights a focused attention process. In contrast, the visual search tasks of Luck et al. (1994) highlight a process of discriminating a target from a number of surrounding distracters, something that does not necessarily require focused attention but instead, a process of monitoring effects in the environment so that attention can essentially *capture the sensory effects* which are relevant for further processing, and notably, the actual response characteristics (response time and accuracy) revealed superiority in the controls (Luck et al., 1994).

The process of “capture” described above is proposed herein as initiating the formation of action-effects codes through association (another rudimentary process). To illustrate further, let us consider a more common example: when a young child first reaches for the television remote and attempts to randomly press its buttons, a salient and novel sensory event will eventually occur – the TV turns on! The child then continues with his button pressing behavior and eventually the event occurs again. With repeated playful (trial-and-error) behavior the child begins to learn that the sensory event (the TV turning on) is associated with his button pressing behavior (action), thereby forming an action-effects perceptual code which, once learned, can be later activated. This process underlying the *formation* of action-effects perceptual codes illustrates Level 1 of the proposed framework. We have suggested that this process is linked to dopaminergic signaling of the basal ganglia system, an idea which has perhaps received the most thorough treatment in the PhD dissertation of Jeffrey Bednark (2012) conducted in my lab and has gained some initial support from earlier animal models (Redgrave and Gurney, 2006) and our recent joint investigations in humans (Bednark, 2012; Bednark et al., 2012).

The basal ganglia are a subcortical complex of nuclei best known for their involvement in Parkinson’s disease which results in severe loss of the key neurotransmitter dopamine within the substantia nigra pars compacta where it is produced, and also in all areas of the brain that receive dopaminergic projections. Involvement of basal ganglia has been implicated in attentive as well as pre-attentive processes (Troscianko and Calvert, 1993; Horowitz et al., 2006; Hayes et al., 1998; Franz and Miller, 2002), and the proposed Level 1 is essentially on the border of those, with salience and novelty of sensory effects capturing the attention of the observer/actor/agent so that additional focused attention can be applied (in order to form an action-effects perceptual code).

Discovering contingencies between sensory effects and the movements that elicit them has been discussed in the context of ideomotor learning with the notion that movements are initially carried out in a predominantly exploratory manner but with the aid of mechanisms such as those underlying the orienting response (Sokolov, 1963) in forming action-effects codes (Elsner and Hommel, 2001, 2004; Herwig and Waszak, 2009; Bednark, 2012). However, a difference in theoretical approach is that ideas underlying ideomotor theory correspond to intentional performance and not more generally the components of an adaptive system of learning which optimizes behavior to salient changes in the environment as in the proposed approach (Franz and McCormick, 2010; Bednark, 2012; Bednark et al., 2012). Extensively studied (although perhaps not in the context of focused attention), the basal ganglia-dopaminergic system forms the basis of numerous highly cited reviews which capture the elegant parallel loop organization of the basal ganglia (striatal)-thalamo-cortical circuits (Alexander et al., 1986) and the manner in which dopaminergic activity modulates synaptic activity intrinsic to the basal ganglia (Bjorklund and Dunnett, 2007) through phasic and tonic signals (Mink, 1996). Those processes are thought to be particularly important in detection of salient sensory signals (perhaps using a superior colliculus input: Comoli et al., 2003; Redgrave and Gurney, 2006) which are often unexpected and in association with rewards (Schultz, 1998). Specifically, the sensory effects are thought to elicit a dopaminergic neuronal response which leads to phasic activity in the striatum of the basal ganglia (Freeman et al., 1985; Horvitz et al., 1997; Schultz, 1998; Bednark, 2012). The proposed Level 1 depends on dopamine input to bind action-effects codes during their initial formation, characterizing a rudimentary learning mechanism and (based on the proposed framework) also a basis on which the development of endogenous focused attention evolved (as Levels 2 and 3). This rudimentary system (Level 1) is conceptualized as one that likely interacts with other subcortical structures including (but not limited to) the cerebellum and hippocampus through cortical loci and with emotion systems through limbic inputs, making up a highly adaptive system of learning (Yin and Knowlton, 2006; Balleine and O’Doherty, 2009; Ashby et al., 2010). Learning occurs at all levels of the proposed framework in increasingly higher degrees of complexity with level of involvement. Thus, Level 1 reflects rudimentary processes of learning upon which Level 2 (and further, Level 3) evolved, and development is proposed to follow a similar trajectory with lower levels becoming embedded into increasingly higher (and more complex) levels in a manner akin to that proposed by Hughlings Jackson (1884); Franz and McCormick, 2010; Franz and Gillett, 2011).

Once the action-effects codes are learned, they must be stored, and this likely involves redundant storage areas of the brain (cerebellum, striatum of the basal ganglia, hippocampus, and cortex). Evidence has shown that both the cerebellum and basal ganglia contain maps of the body (Hoover and Strick, 1999), which is consistent with storage of lower level codes, and cortical networks are likely to be involved in higher level storage as well. The key processes of activation and inhibition proposed to occur initially in Level 2 (but also in higher levels) are also thought to be the basis of sequencing and switching of perceptual codes which can be described as involving associative processes built upon rudimentary functions of Level 1 (see Figure 1).

Supporting the activation-inhibition processes of Level 2, is a basic model in which the intrinsic circuits of the basal ganglia result in the activation of desired actions and inhibition of competing and prepotent actions (Mink, 1996; Franz, 2006). This model derives from the classic model of Parkinson’s disease in which the hallmark symptoms (e.g., tremor, dyskinesia, akinesia) are proposed to be due to an imbalance of the direct and indirect circuits which comprise the primary architecture of the basal ganglia system, with such a balance contributing to aggregate effects of disinhibition on the thalamic nuclei that receive basal ganglia output to enable for desired motor responses without interfering effects of irrelevant or undesired prepotent responses (Mink, 1996; Franz, 2006). Evidence consistent with this model comes from experimentation on simple responses (Franz and Miller, 2002) and other motor and cognitive processes (Hayes et al., 1998; Shook et al., 2005; Franz, 2006) in people with Parkinson’s disease. For example, Franz and Miller (2002) tested people with Parkinson’s disease and controls matched for a number of demographic parameters on simple responses using manipulated levels of response readiness (Franz and Miller, 2002). A color pre-cue indicated that a Go signal would follow on 80% of trials (high readiness), and a different color pre-cue indicated that a Go signal would follow on only 20% of trials (low readiness), with No-Go signals on the remaining trials in each case. Results revealed that whereas the same patterns occurred in the two groups in terms of the influence of response readiness on reaction time (RT; i.e., higher levels of response readiness leading to faster RTs compared to low levels of response readiness), this consistent pattern across groups was not found for the force impulse which was modulated with level of response readiness in controls but not in people with Parkinson’s disease. This led to the interpretation that there is a basic impairment in processes related to conversion of levels of response readiness to appropriate activation of responses (Franz and Miller, 2002). Interestingly, Sperry (1952, p. 302) alluded to something similar in his earlier suggestion that the function of *perception* might be “an implicit preparation to respond… to prepare the organism for adaptive action.” Other evidence from Franz and Miller (2002) revealed impairments related to inhibition, with inappropriate levels of force occurring in people with Parkinson’s disease, for example, in the hand that was not supposed to respond on a trial, which seems to be the flip-side of activation, fitting well with the framework of Mink (1996) in which the direct circuit increases activation whereas the indirect circuit increases activity in the opposite direction (inhibition). Thus, the proposed Level 2 of the framework described herein is characterized by rudimentary processes of activation and inhibition of action-effects perceptual codes ranging from elemental ones (as in the case of Franz and Miller, 2002), to those making up perceptual sets (Shook et al., 2005) and other more complex associations (Franz, 2006). Activation and inhibition processes built upon those attributed to Level 2 are further elaborated at Level 3 which involves increasing cortical influences that are part of the highly evolved basal ganglia-thalamic-cortical circuits and the involvement of cortical networks depending on the specific nature of the information. Moreover, thalamic feedback loops with the striatum (the input nucleus of the basal ganglia) have more recently been suggested as modifying the classical model of direct and indirect circuits (that of Mink, 1996), suggesting a more elaborated model (McFarland and Haber, 2000). Furthermore, as suggested above, the rudimentary processes of association, activation, and inhibition as proposed in Levels 1 and 2 are also involved (perhaps in elaborated and higher levels of processing) in tasks such as sequence learning (e.g., pre-SMA: Sakai et al., 1999), and task switching (particularly implicating prefrontal regions in addition to the basal ganglia: Benecke et al., 1987; Hayes et al., 1998; Cools et al., 2001; Shook et al., 2005).

It is herein proposed that with learning, focused attention essentially shifts from the elemental components (basic action-effects perceptual codes) to higher-order codes associated with sequences or chunks, and in the interest of efficiency, perceptual codes no longer adaptive are replaced with those that are, with a highly flexible allocation of attention. Moreover, perceptual codes for highly automatized routines require less focused attention to activate than would a series of codes associate with more elemental movements. The flexible allocation of this proposed system therefore depends on many factors related to the task demands and situation (bottom-up and top-down), but also on response dependencies and unifying constraints (such as those earlier described). In this manner, the so-called hierarchy of goals (and subgoals) of Cooper and Shallice (2006) is captured primarily in Level 3 which serves to alter the organization and content of stored action-effects perceptual codes. Level 3 also incorporates processes of learning in which goal-directed actions become habits (Ashby et al., 2010), reflecting the proposed “automaticity loop” (upper right corner of Figure 1). Thus, the fundamental functions of capture and association (from Level 1), and activation and inhibition (from Level 2) are further incorporated into higher-order levels of action-effects perceptual codes (e.g., Level 3), due to the propensity for unifying representations at all levels of the highly adaptive system (Franz and McCormick, 2010).

As pointed out earlier, research in the normal brain suggests that the two hemispheres usually tend to work together to produce a common goal or conceptually unified action or thought (Franz et al., 2001; Franz, 2010; Franz and McCormick, 2010). Thus, the goal-relatedness of a sequence of perceptual codes must also be learned, and this often occurs with very high levels of cognition such as that involved in language (Franz and McCormick, 2010), with further processes of learning serving to alter the hierarchical organization of the perceptual codes (Cooper and Shallice, 2006).

One might speculate (as we did), that the intact corpus callosum would be highly necessary for the learning of goal-directed actions, particularly those involving both sides of the body (e.g., bimanual) and/or the binding of movement sequences with speech-word labels. Franz et al. (2000) provides strong experimental support of this prediction. People with commissurotomy were asked to wear a blindfold (to prevent input from going to both hemispheres via the visual modality) and to pantomime actions of the hands in response to experimentally manipulated verbal commands in the form of unimanual actions like “grab an orange” or bimanual actions like “peel an orange.” The trials of key interest were the bimanual ones in cases of novel or familiar actions. Results revealed that if the actions were novel in that they were never before performed by the participant, the pantomimed gestures appeared as mere random movements that did not resemble the “commanded” actions in any way. It was only when the actions were well-learned prior to commissurotomy that the participants could pantomime them relatively accurately (and note that the verbal command was available to both cerebral hemispheres which are both capable of understanding verbal descriptions; Franz et al., 2000). An intriguing (albeit modest) dissociation is that one commissurotomy participant who frequently went fishing could pantomime the action of “putting a hook on a line” although he could not pantomime the similar motor procedures involved in the action of “threading a needle,” claiming “I don’t sew!” To the converse, a second commissurotomy participant was easily able to pantomime the action of “threading a needle” when asked to, but could not “put a hook on a line” stating clearly “I have never fished!” One might infer that the elemental response codes of the actions would be similar in the two cases even though their verbal labels are different. Indeed, the well-learned elemental movement sequences were intact and could be retrieved somewhat automatically (reflecting successful learning based on Level 2 processes). However, those actions that had never been learned (i.e., novel actions) could not be performed upon verbal command. Those are proposed to depend on Level 3 for the high levels of learning required in the formation of complex action-effects codes in which the (verbal/speech) goals are also bound to the pantomimed actions. Findings of Franz et al. (2000) also support the framework that higher elaborated levels of the sensorimotor system (those related to representations of goal-directed actions) override and/or inhibit lower sensorimotor networks, a property of Level 3 which assists in modulating lower level systems and enabling the two hands to work cooperatively to achieve a common goal (Franz and McCormick, 2010). Notably, the well-learned actions were still readily retrievable in the participants with commissurotomy (Level 3) again pointing to subcortical processes as being integral (Franz et al., 2000); although as alluded to earlier, it is outside of the present framework to describe precisely where and how those codes are stored. As described above, focused attention is conceptualized as being flexibly allocated in accordance with the level of network involvement such that *activation of high level networks* is virtually synonymous with *attention to high level action-effects codes (*which also contain the verbal codes corresponding to goals*)*. The flexible allocation of attention to goals within a hierarchical representation also seems consistent with the Action Identification Theory of Vallacher and Wegner (1989) in research on individual differences and personality.

According to the proposed account, in learning of complex actions our brains resort to the most economical form of action guidance, and that is to be aware of (conscious of) the high level action goals (i.e., threading a needle), while (with learning) becoming less aware of (conscious of) the details of the procedures to achieve the action goal (i.e., moving one hand upward to hold the needle while moving the other hand horizontally to thread it). Through learning, our focused attention essentially moves to different networks of processing from those that were initially involved in first learning the elemental movements. Thus, our action goals involve processing (or *attention*) at higher-order networks, and the precise movement procedures are unattended once actions are learned (Franz, 2010; Franz and McCormick, 2010). We can easily confirm this basic model by asking ourselves after the fact of learning – “what did I just do?” The answer – “I threaded a needle.” The elemental responses used in that well-learned action are not what we tend to recall unless specifically asked to, and even then, it takes quite a lot of effortful and imagery-laden processing to do so.

## Distinguishing Bilateral Projections of Sensory and Motor Input-Output Systems from the Subcortical System Proposed Herein

Gazzaniga and Sperry (1967) noted that “The disruption of interhemispheric integration produces remarkably little disturbance in ordinary daily behavior, temperament, or intellect. The functional deficits tend to be compensated by the development of bilateral motor control from each hemisphere, also by bilaterality in some of the sensory projection pathways and by a variety of other unifying factors that we deliberately avoid or exclude so far as possible in the testing procedures” (p. 131–132). The upper distal extremities (and the speech system) are usually referred to in association with lateralized control, in comparison to the proximal extremities (bilateral control). Sperry also pointed out other bilateral projections which include the cutaneous sensory system for the face which is mediated by the trigeminal nerves for each side represented in both hemispheres. As already stated, audition is also bilateral. So are systems mediating crude pain, temperature, pressure, and position sense, especially from axial parts of the body, and exploratory movements of the eyes can provide bilateral representation of a scene (Sperry, 1986). Mental-emotional ambiance or semantic surround in self and social awareness also spreads quickly to both hemispheres (Sperry, 1986).

Decades ago Sperry also noted “The extrapyramidal motor outflow from the cerebral cortex likewise arises from associative and sensory cortical fields as well as from those traditionally designated as motor. Excitation patterns in the sensory and associative areas, therefore, have to integrate with patterns in the subcortical motor systems as well as with those in neighboring motor fields” (Sperry, 1952, p. 300). It is these which in my view implicate the subcortical processes I am referring to as comprising the basic components of the allocation of attention system. Furthermore, unifying constraints are conceptualized as operating in a ubiquitous manner at all levels of the system, primarily by reducing processing demands, and their associated networks are highly plastic, i.e., they change with learning. In contrast, bilateral projections are hardwired although they are modified through developmental processes such as myelination (Paus et al., 1999) and synapse and/or axonal elimination (Purves and Lichtman, 1980; for reviews see Blakemore and Choudhury, 2006; Turney and Lichtman, 2012).

The proposed account (of a basal ganglia-thalamic-cortical system) underlying the system of allocation of attention is intended to apply for a vast range of actions which include multi effector and bimanual actions (in addition to unimanual actions). Thus, in the case of actions employing effectors controlled by two different hemispheres, although learning of new actions (in association with high level codes) cannot occur without the corpus callosum, learning at lower levels of the system together with the capability to retrieve well-learned action-effects codes, would still be possible (Franz et al., 2000). Evidence from animal and human studies reviewed recently in Brun et al. (2012) including findings of their own, reveals that unilateral manipulations result in bilateral activity in the striatum (the primary input nucleus of the basal ganglia), and other nuclei of the basal ganglia including the globus pallidus externus (GPe), subthalamic nucleus (STN), and the globus pallidus internus (GPi), a primary output nucleus of the basal ganglia. Evidence reveals, for example, that high frequency stimulation to the unilateral STN results in complex changes in bilateral basal ganglia-thalamic-cortical networks. According to a study by Parent et al. (1999), the GPi projection in primates reveals ipsilateral and contralateral projections to the thalamus and brainstem, based on the use of retrograde cell labeling techniques. In addition, as alluded to earlier, evidence suggests that the classical model involving direct and indirect circuits (Mink, 1996) requires modification, particularly on the basis of Parent and Parent (2002) who report a high degree of axonal collateralization in the system, and McFarland and Haber (2000) who report not only that striatal output projects to thalamic nuclei (the traditional model), but also, that ventral thalamic nuclei relay input back to the dorsal striatum. Furthermore, there appears to be a convergence of information onto the dorsal striatum (Takada et al. (1998a,b)), which is consistent with the notion of “unifying constraints” proposed herein (although this is a loose analogy). Together, this evidence suggests that the striatum of the basal ganglia is part of a complex and widely distributed network that can influence other neural areas in complex ways (including bilaterally), and the thalamus feeds back information to the striatum, thereby further influencing that system. However, as those researchers also state, it is not yet known precisely how the basal ganglia circuitry influences neural activity bilaterally, nor which precise circuits are involved. The hope (and prediction) is that future research will further elucidate the precise bilateral projections involved. Interestingly also, the neuroanatomical findings reveal a hierarchical structure of the basal ganglia system (references above), consistent to what is described herein.

## Evidence of Very High Level Constraints Derived from Our Social Interactions

We have learned from experimentation on the split-brain, that the capacity for focused attention is limited although unified (Kreuter et al., 1972) even though responses can be lateralized, although it is not fully understood how unity of behavior occurs in a brain with lateralized function. However, one other clue might be worth considering. A mechanism was earlier proposed in the interest of alerting one hemisphere that a distal manual response is pending in the other, and depends on the integrity of the primary cortical commissure, the corpus callosum (Franz and Fahey, 2007). However, unlike for the manual system there is no proposed alerting mechanism for the speech system, and while the hands often work together to satisfy a common goal with higher levels overriding lower levels of the system (Franz and McCormick, 2010) this does not apply in the same way for speech. Furthermore, it would be disastrous for unified behavior if two different speech outputs occurred simultaneously in the different hemispheres! It appears that a unique form of complementarity has evolved in facilitating social interactions in humans. We speak to others, yet we also listen, observe, and sometimes even touch. We often perceive others’ expressions, intonations, and bodily movements while we speak. In fact, very clever research studies have shown that our understanding of speech is greatly facilitated by concurrent observations of facial gestures of those we are conversing with (i.e., listening to), a finding stemming from the so-called McGurk and MacDonald (1976) effect (Baynes et al., 1994; Gentilucci and Cattaneo, 2005; Rosenblum, 2010). So many of the other functions which have shown some lateralized hemisphere specializations seem to be related in some way to this basic dichotomy of speech and facial expression recognition. Evidence from studies using chimeric displays point to a right hemisphere superiority in facial emotion, as do closely related functions such as those involved in processing spatial relations of objects, geometric patterns, and various other familiar or unfamiliar stimuli (Levy et al., 1972; Bourne, 2008). Additional evidence of this right hemisphere superiority comes from part-whole and gestalt completion tests (Nebes, 1974), geometric tasks (Franco and Sperry, 1977), mental rotation (Corballis and Sergeant, 1988), visuo-spatial construction (Sperry et al., 1969), recognition of objects, scenes, and personally relevant people (Sperry et al., 1979), and in general, processes associated with appreciating communicative significance and emotional tone (Sperry, 1977) of prosody and bodily postures (George et al., 1996; Downing et al., 2001). In contrast, tasks which reflect forms of symbolic processing such as numerical calculations, the ordering and sequencing of events (which often are associated with speech; Gazzaniga and Sperry, 1967; Kreuter et al., 1972), and reading and writing (Sperry, 1977), all could be construed as stemming from the basic function of speech. Thus, speech interactions which form a dominant form of social interaction, might have led to a unique form of unity in behavior comprised of a complementarity in the division of labor of the two hemispheres. Evidence from the “split-brain” clearly illustrates this unique form of complementarity which combines unifying constraints at a very high level together with response dependencies.

## Uniqueness of the Present Framework

The present framework proposes a multilevel system for the allocation of attention for action, in which the dopaminergic basal ganglia-thalamic-cortical circuits are integral. Although the present framework shares some features with models in the literature (as pointed out above), it also reflects a number of unique aspects. Notably, the present framework builds upon a highly dynamic system in which subcortical processes are central to the networks involved, therefore proposing a different, or perhaps, complementary approach to localization models in which the primary focus is cortical networks (particularly in the case of sequencing, e.g., Keele et al., 2003). The present framework is based on an embodied cognition view in which cognition evolved from basic sensorimotor interactions with the environment, although symbolic representations are thought to build upon those rudimentary forms of representations (Franz and McCormick, 2010, footnote 1). The present framework is based on an evolutionary model in which higher cortical processes evolved from rudimentary functions of primarily subcortical systems, with further evolution of increasingly higher levels, with the involved cortical levels linked to the nature of information being processed. The present framework also illuminates what is proposed to be the basic processes (capture, association, activation, inhibition) on which procedural learning is built, with the idea that sequencing and switching are elaborated forms of those rudimentary processes, as might be “selection” when considering further influences of top-down processes. Furthermore, this framework defines “representation” as the action-effects perceptual codes underlying action learning and memory in general, further exemplifying an embodied system. As pointed out above, the synthesis of experimental evidence from cognitive studies, a neural model involving the basal ganglia-thalamic-cortical system, and a framework of allocation of attention to action, is original, to the author’s knowledge. Although an attempt is made to point out relevant overlap with existing models in the literature, space limitations prohibit the inclusion of other very important and relevant research that also bears on the topic of this paper. For example, specific findings in the area of Parkinson’s disease are left for a companion review.

## Summary and Conclusion

The proposed account is summarized as follows: a highly evolved system of attention allocation depends on the integrity of subcortical-cortical circuits of which the basal ganglia complex and thalamus are at the very heart; other subcortical networks and structures outside of the scope of the present review are undoubtedly involved but are not described in this paper. A highly flexible system regulates a dynamic allocation of focused attention to the formation, activation, inhibition, and selection of action-effects perceptual codes which form a hierarchical organization with increasingly higher levels reflecting learning. This proposed system operates in the intact brain and the brain with commissurotomy. However, the “split-brain” does not have the normal integration of spatial processes between the two cerebral hemispheres, nor does it have the so-called manual alerting system (described herein); furthermore, the mechanism proposed to assist in equilibrating attention across the hemispheres (Kreuter et al., 1972) might actually rely largely on the basal ganglia-thalamic-cortical system described herein. Our social interactions, using our most dominant response mode – that of speech – reveal a primary response dependency which shapes our conscious experience by imposing limitations on the availability of information to processing networks (i.e., the non-speech hemisphere in the case of the “split-brain”). This type of response dependency also illustrates a unique division of labor in which complementarity of functions in the two cerebral hemispheres evolved as a unifying constraint at very high levels of the system, building on the propensity of the brain to process information in a unified manner at all levels of perception, thought, and action (Franz and McCormick, 2010).

In closing, it is perhaps important to again point out that focused attention and conscious experience are not necessarily the same thing according to careful analysis based on processing of vision (Lamme, 2003). However, the present framework suggests that what becomes part of conscious experience can be strongly influenced by response dependencies and ultimately the action-effects codes we attend to.

## Conflict of Interest Statement

The author declares that the research was conducted in the absence of any commercial or financial relationships that could be construed as a potential conflict of interest.
